# Case report of an atypical early onset X-linked retinoschisis in monozygotic twins

**DOI:** 10.1186/s12886-017-0406-6

**Published:** 2017-02-24

**Authors:** Vittoria Murro, Roberto Caputo, Giacomo Maria Bacci, Andrea Sodi, Dario Pasquale Mucciolo, Sara Bargiacchi, Sabrina Rita Giglio, Gianni Virgili, Stanislao Rizzo

**Affiliations:** 1 Department of Translational Surgery and Medicine, Eye Clinic, University of Florence, Largo Brambilla, Florence, 3-50134 Italy; 2 Ophthalmology Unit, Department of Pediatrics, Anna Meyer Children’s University Hospital, Florence, Italy; 3 Genetics and Molecular Medicine Unit, Department of Pediatrics, Anna Meyer Children’s University Hospital, Florence, Italy

**Keywords:** X-linked retinoschisis, Juvenile retinoschisis, *RS1*, Bullous peripheral schisis, Case report

## Abstract

**Background:**

X-linked Retinoschisis (XLRS) is one of the most common macular degenerations in young males, with a worldwide prevalence ranging from 1:5000 to 1:20000. Clinical diagnosis of XLRS can be challenging due to the highly variable phenotypic presentation and limited correlation has been identified between mutation type and disease severity or progression.

**Case presentation:**

We report the atypical early onset of XLRS in 3-month-old monozygotic twins. Fundus examination was characterized by severe bullous retinal schisis with pre-retinal and intraretinal haemorrhages. Molecular genetic analysis of the *RS1* was performed and the c.288G > A (p. Trp96Ter) mutation was detected in both patients.

**Conclusions:**

Early onset XLRS is associated with a more progressive form of the disease, characterized by large bullous peripheral schisis involving the posterior pole, vascular abnormalities and haemorrhages. The availability of specific technology permitted detailed imaging of the clinical picture of unusual cases of XLRS. The possible relevance of modifying genes should be taken into consideration for the future development of XLRS gene therapy.

## Background

XLRS is one of the most common macular degenerations in young males with a worldwide prevalence ranging from 1:5000 to 1:20000 [1]. The clinical hallmark of the disease is a spoke-wheel pattern foveal schisis associated with a peripheral schisis in about 50% of affected patients. Foveal involvement, which is present in all affected patients, is usually associated with gradual progression of visual loss [2]. Molecular genetic studies identified the *RS1* gene on chromosome Xp22 as cause of the disease [3]. This gene codes for a protein named retinoschisin that is implicated in cellular adhesion and cell–cell interactions. The function of retinoschisin in the retina is not well understood, but it is thought to be involved in cell adhesion maintaining the integrity of the photoreceptor-bipolar synapse. Another possible role of retinoschisin is the regulation of cellular fluid balance, and its lack could be the cause of pathological extracellular fluid accumulation in the form of cystic cavities [2]. Although diagnosis is often delayed until school age, a few cases have been described in the first year of life [4–6] suggesting that XLRS could be present even at birth. Clinical diagnosis of X-linked retinoschisis can be challenging due to the highly variable phenotypic presentation [2] and limited correlation has been identified between mutation-type and disease severity or progression [7, 8]. We report the atypical early onset of XLRS in 3-month-old monozygotic twins.

## Case presentation

Three-month-old monozygotic male twins (Twin-A and Twin-B) arrived at the Pediatric Ophthalmology Department of the Anna Meyer Children’s Hospital for a follow-up examination due to mild prematurity and bilateral esodeviation of the eye-globes. They were born at 34 weeks and their weight was 2300 and 2400 g respectively. Family history was negative for ocular diseases.

**Fig. 1:**
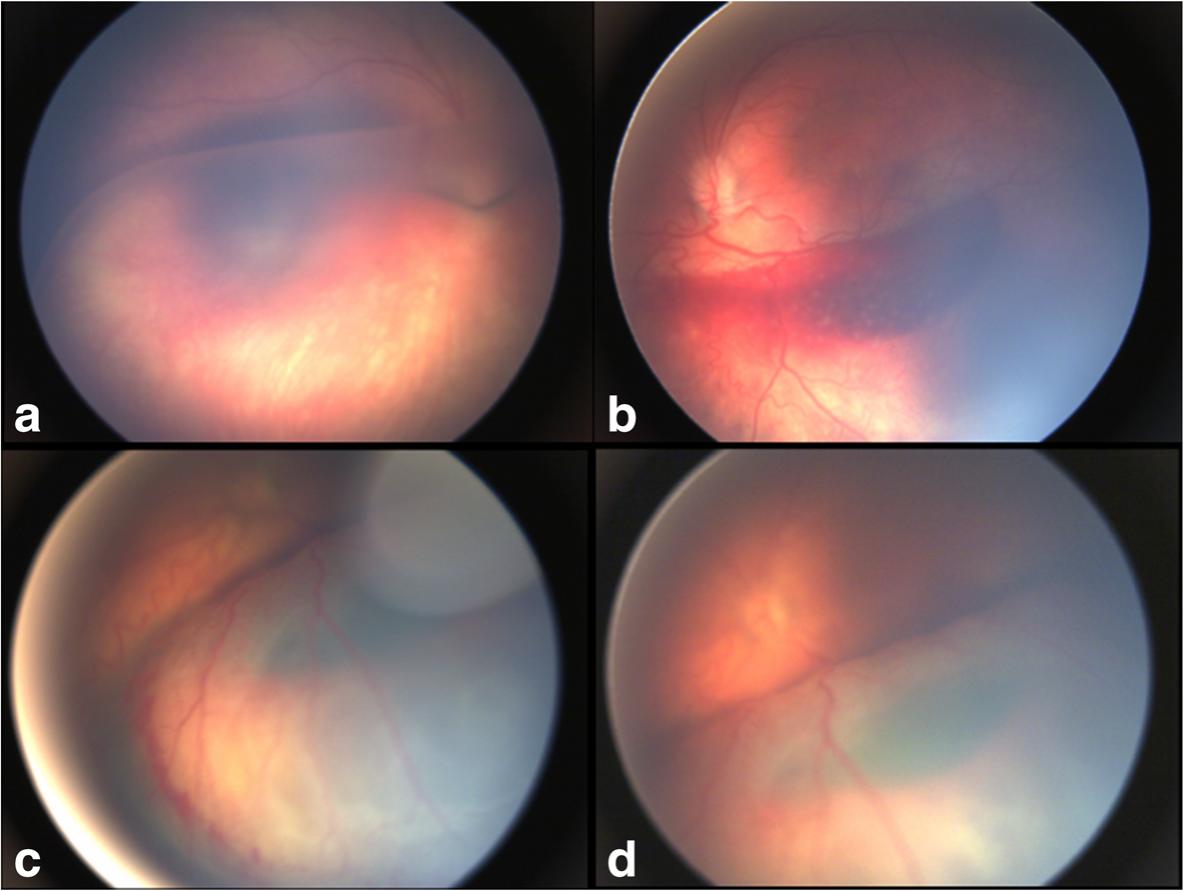
**a** The peripheral schisis covers the macular area in the right eye of Twin-A; **b** An intraretinal haemorrhage is present within the inferior schisis in the left eye of Twin-A; **c** Pre-retinal haemorrhages are clearly evident on the surface of the schisis in the right eye of Twin-B; **d** A haemorrhage can be seen deep in the retina in the right eye of Twin-B

**Fig. 2:**
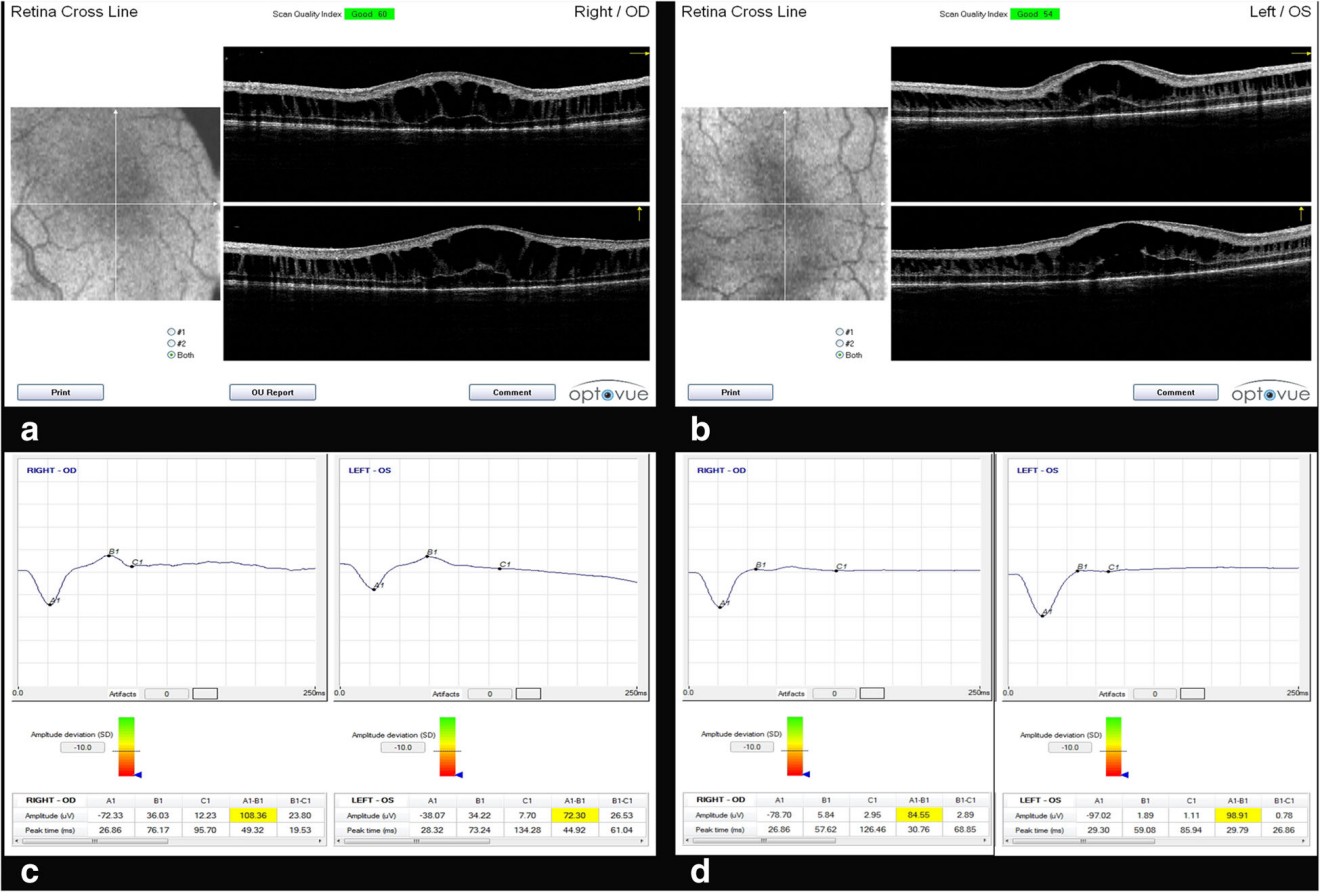
The OCT scans (**a**,**b**) show a macular schisis involving different retinal layers (inner nuclear, outer plexiform and outer nuclear layer) which extends beyond the foveal area, in the right and left eye of twin-B; The ERG plot (**c**,**d**) shows an absent b-wave and a nearly normal a-wave (negative ERG). **c** right and left eye of twin-A with b/a wave ratio 0,49 and 0,89 respectively; **d** right and left eye of twin B with b/a wave ratio 0,07 and 0,02 respectively. The background luminance is 30 Cd/m2; Flash strength 3.000 Cds/m2; flash frequency 30.00 (Hz)

On examination, the anterior segment was unremarkable. In Twin-A fundus examination of the right eye revealed a severe schisis reaching the posterior pole and preventing visualization of the fovea (Fig. 1a), while in the left eye the inferior schisis was complicated by an intra-retinal haemorrhage (Fig. 1b). Twin-B showed in the right eye a severe bullous peripheral schisis with pre-retinal (Fig. 1c) and intra-retinal haemorrhages (Fig. 1d); the left eye showed a schisis from the inferior quadrant shrouding to the posterior pole. Fundus pictures were acquired using the RetCam (Clarity Medical Systems Inc., Pleasanton, CA) and optical coherent tomography (OCT) scans were then obtained using the I-Vue Hand Held Spectral Domain - OCT (Optovue Fremont, CA). In the right eye of Twin-A the size of the bullous elevated schisis prevented foveal visualization and OCT investigation. In the remaining 3 eyes OCT scans revealed macular schisis with intraretinal separation in different retinal layers (intraretinal cysts could be seen in the inner nuclear, outer plexiform and outer nuclear layer), extending beyond the foveal area. In Fig. 2 (Fig. 2a,b) we have shown OCT scans from the right and left eyes of Twin-B. The patients were examined under general anaesthesia after obtaining informed consent from the parents.

Finally the Electroretinogram (ERG; Retimax CSO Firenze Italy) was recorded according to the International Society for Clinical Electrophysiology of Vision (ISCEV) standard [9]. In all the 4 eyes ERG showed the typical absence of the b-wave and a nearly normal a-wave (negative ERG) (Fig. 2 c,d).

Molecular genetic analysis of the *RS1* was performed because diagnostic criteria for XLRS was present. It detected in both patients the c.288G > A (p.Trp96Ter) mutation.

## Discussion and conclusions

At present detailed imaging of early XLRS onset in infant twins cannot be found in literature, both because of the rarity of such clinical cases and because in very young babies retinal imaging requires specific technology not always available. In our patients hand-held Ret-Cam and SD-OCT permitted the early clinical diagnosis of XLRS later confirmed by electrophysiology and molecular genetic testing. The patients reported by Prasad et al. [4] and Lee et al. [6] showed vitreous haemorrhage as the initial feature of the disease, while our patients showed intra-schisis haemorrhages and retinal vascular abnormalities without vitreous haemorrhage at the time of diagnosis. All these cases show a severe phenotype suggesting that early onset is probably associated with a more progressive form of the disease although there are some differences in the clinical aspects.

To our knowledge, only one other case of XLRS in monozygotic twins has previously been reported [10] within a relatively large series of 14 patients. The study was mainly a molecular genetic investigation and so it provided poor details concerning the clinical features of the patients and the phenotype similarities between the twins.

In our patients molecular genetic analysis supported the diagnosis of X-linked retinoschisis by detecting in both twins the *RS1* sequence variant c.288G > A (p. Trp96Ter). This mutation is a stop mutation, leading to a truncated polypeptide. It is located in the discoidin domain of the protein, a crucial region for its functional activity; for these reasons it is likely pathogenic and moreover this mutation has already been associated with the disease [11]. XLRS is usually characterized by a high degree of phenotypic variability, even within the same family [2]. The three XLRS male patients of the same family with *RS1* variant c.288G > A (p.Trp96Ter), previously reported in literature [11] showed peri-foveal radial micro-cysts and a silver-grey reflex in the peripheral retina. None of them presented peripheral retino-schisis. Furthermore they were characterized by a variable degree of visual dysfunction, in fact the younger boy (5 years of age) showed poor visual acuity, while his cousin (38 years of age) had vision that was good enough to allow him to acquire a driver’s license. On the contrary, our twin patients, despite having the same mutation as them [11], share a more severe phenotype with very similar clinical features (very early onset of the macular abnormalities, large peripheral retino-schisis involving the posterior pole and intra-schisis hemorrhages).

As regards the relationship between the XLRS genotype and phenotype, our clinical case allows us to speculate a key role of some modifier genes, which are involved, in particular, in twins. Furthermore, the early onset of the disease minimises the contribution of environmental factors, underlining that the phenotype results from the interaction of various genes, not only the disease gene. This consideration may have important implications for gene therapy. Currently two Phase I/II clinical trials of intra-vitreally delivered *RS1* for X-linked retino-schisis (ClinicalTrials. gov identifiers: NCT02317887 and NCT02416622) are underway thanks to the results of the preclinical gene delivery of human *RS1* in a mouse-model of the disease. [12]. The results that will be obtained in the future will further clarify our understanding of this disease and its genotype/phenotype relationship and the selection criteria for the gene therapy.

In conclusion we describe a case of monozygotic twins affected by early onset severe phenotype of X-linked retinoschisis. The availability of specific technology permitted detailed imaging of the clinical picture of this unusual case. The possible relevance of modifying genes should be taken into consideration for the future development of XLRS gene therapy.

## Abbreviations

ERG: Electroretinogram
ISCEV: International Society for Clinical Electrophysiology of Vision
OCT: Optical coherent tomography
XLRS: X-linked Retinoschisis

## References

[1] George ND, Yates JR, Moore AT (1995). X linked retinoschisis. Br J Ophthalmol.

[2] Molday RS, Kellner U, Weber BH (2012). X-linked juvenile retinoschisis: clinical diagnosis, genetic analysis, and molecular mechanisms. Prog Retin Eye Res.

[3] Sauer CG, Gehrig A, Warneke-Wittstock R, Marquardt A, Ewing CC, Gibson A, Lorenz B, Jurklies B, Weber BH (1997). Positional cloning of the gene associated with X-linked juvenile retinoschisis. Nat Genet.

[4] Prasad A, Wagner R, Bhagat N (2006). Vitreous hemorrhage as the initial manifestation of X-linked retinoschisis in a 9-month-old infant. J Pediatr Ophthalmol Strabismus.

[5] Renner AB, Kellner U, Fiebig B, Cropp E, Foerster MH, Weber BH (2008). ERG variability in X-linked congenital retinoschisis patients with mutations in the RS1 gene and the diagnostic importance of fundus autofluorescence and OCT. Doc Ophthalmol.

[6] Lee JJ, Kim JH, Kim SY, Park SS, Yu YS (2009). Infantile vitreous haemorrhage as the initial presentation of X-linked juvenile retinoschisis. Korean J Ophthalmol.

[7] Pimenides D, George ND, Yates JR, Bradshaw K, Roberts SA, Moore AT, Trump D (2005). X linked retinoschisis: clinical phenotype and RS1 genotype in 86 UK patients. J Med Genet.

[8] Vincent A, Robson AG, Neveu MM, Wright GA, Moore AT, Webster AR, Holder GE (2013). A phenotype-genotype correlation study of X-linked retinoschisis. Ophthalmology.

[9] McCulloch DL, Marmor MF, Brigell MG, Hamilton R, Holder GE, Tzekov R, Bach M (2015). ISCEV standard for full-field clinical electroretinography (2015 update). Doc Ophthalmol.

[10] Hotta Y, Fujiki K, Hayakawa M, Ohta T, Fujimaki T, Tamaki K, Yokoyama T, Kanai A, Hirakata A, Hida T, Nishina S, Azuma N (1998). Japanese juvenile retinoschisis is caused by mutations of the XLRS1 gene. Hum Genet.

[11] Sato M, Oshika T, Kaji Y, Nose H (2003). Three novel mutations in the X-linked juvenile retinoschisis (XLRS1) gene in 6 Japanese patients, 1 of whom Had Turner’s syndrome. Ophthalmic Res.

[12] Min SH, Molday LL, Seeliger MW (2005). Prolonged recovery of retinal structure/function after gene therapy in an Rs1hdeficient mouse model of x-linked juvenile retinoschisis. Mol Ther.

